# Are We Chasing a Wild Goose? Rethinking Breeding Targets for Salinity Stress Tolerance in Rice

**DOI:** 10.3390/plants15040597

**Published:** 2026-02-13

**Authors:** Qian Xu, Ping Yun, Kiril Tenekedjiev, Natalia Nikolova, Babar Shahzad, Jiarui Zheng, Lana Shabala, Meixue Zhou, Sergey Shabala

**Affiliations:** 1 School of Architecture and Planning, Foshan University, Foshan 528000, China; xuqian@fosu.edu.cn; 2 International Research Centre for Environmental Membrane Biology, Foshan University, Foshan 528000, China; 3 School of Biological Science, University of Western Australia, Perth, WA 6009, Australia; ping.yun@uwa.edu.au (P.Y.); l.shabala@utas.edu.au (L.S.); 4 Australian Maritime College, University of Tasmania, Launceston, TAS 7250, Australia; kiril.tenekedjiev@utas.edu.au; 5 Department of Computer Sciences, Varna Free University “Chernorizets Hrabar”, 9007 Varna, Bulgaria; nataliya.nikolova@vfu.bg; 6 Department of Information Technology, Nikola Vaptsarov Naval Academy, 9002 Varna, Bulgaria; 7 School of Agriculture, Food & Wine, University of Adelaide, Adelaide, SA 5005, Australia; babar.shahzad@adelaide.edu.au; 8 Hubei Key Laboratory of Spices & Horticultural Plant Germplasm Innovation & Utilization, School of Horticulture and Gardening, Yangtze University, Jinzhou 434023, China; jiarui.zheng@yangtzeu.edu.cn; 9 Tasmanian Institute of Agriculture, University of Tasmania, Hobart, TAS 7005, Australia; meixue.zhou@utas.edu.au

**Keywords:** salt stress, rice, salt tolerance, K^+^ retention, Na^+^ exclusion

## Abstract

Salinity stress has become an increasingly critical challenge for agricultural production, especially for rice, a staple crop that feeds over 50% of the world population but is extremely sensitive to salt stress. In this study, ten rice genotypes were treated with three salinity levels (0, 50, and 100 mM NaCl) to investigate the effects of salt stress on rice, and this data was then used to build regression models that describe plant growth responses as a function of stomatal conductance (Gs), chlorophyll content (SPAD), and shoot K^+^ and Na^+^ contents—parameters that can be used for high-throughput screening of rice plants for salinity stress tolerance. In silico modeling results showed that the best model for predicting shoot dry weight (SDW) was based on Gs, SPAD, and shoot K^+^ content, while shoot Na^+^ content had no significant influence on biomass accumulation. These findings challenge the traditional focus on Na^+^ exclusion from the shoot as a breeding target and suggest that enhancing K^+^ retention and optimizing stomatal development and operation may be a more effective strategy for improving rice growth under salinity. Overall, this study highlights the need to reconsider key genetic targets involved in the regulation of Gs, K^+^ homeostasis, and chlorophyll maintenance to better face the challenges caused by salinity in future climate scenarios.

## 1. Introduction

Salinity has become one of the most severe threats to global agricultural production, with more than one-third of irrigated land being destroyed by salinization [1]. The issue has worsened due to climate change, excessive irrigation and improper drainage, and other contributing factors [2,3]. As all modern crops are classified as glycophytes, the trend toward land salinization poses a significant threat to global food security. For example, rice is a major staple food for billions of people, accounting for 50 to 80% of their daily caloric intake but is highly sensitive to salinity. Even modest salinity levels of around 4 dS/m (considered as a salinity threshold) may cause yield declines of over 50% in rice, while the predicted levels of soil salinization by 2050 could make rice production simply unfeasible, given current climate scenarios [1].

Many attempts have been made to develop rice varieties with improved salt tolerance. However, these efforts have always faced challenges. Most efforts were focused on a single objective: reducing Na^+^ accumulation in the shoots. To this end, the major strategy has been to enhance Na^+^ exclusion mechanisms, especially those mediated by HKT1 transporters [4,5,6], under the assumption that minimizing tissue Na^+^ would result in higher tolerance. The HKT family can be divided into two distinct sub-families based on their ion selectivity [7], and transporters in sub-family I, such as TmHKT1;5 in wheat, AtHKT1;1 in *Arabidopsis*, or OsHKT1;1 and OsHKT1;5 in rice, are highly selective for Na^+^ [8] and operate in Na^+^ retrieval from the shoot. In the rice genome, a total of nine HKT genes have been found, divided between subfamilies I and II [9]. One of them, OsHKT1;5, has been narrowed down as a determinant of salt tolerance in rice by QTL analysis and attributed to the SKC1 locus [10]. However, some independent studies questioned the validity of this report, showing that NIL(SKC1) plants possessed a more sensitive phenotype (e.g., a larger proportion of chlorotic and necrotic leaves and significantly lower relative shoot and root dry weights) compared to WT [11]. This is hardly surprising, as the strategy of excluding Na^+^ from uptake comes with a caveat of a compromised osmotic adjustment [12,13] and will inevitably yield penalties associated with the high cost of de novo production of organic osmolytes. Also, more recent studies suggest that the primary function of HKT1 is not only to exclude Na^+^ from leaves but also to protect reproductive tissues from Na^+^ toxicity, which can sometimes be achieved even with high Na^+^ accumulation in leaves [14]. Interestingly, plants with impaired HKT1 function that consequently exhibit elevated shoot Na^+^ levels can still exhibit salt tolerance, implying the presence of effective tissue tolerance mechanisms [14]. Taken together, this evidence highlights a critical flaw in the exclusion-centric model and emphasizes the urgent need to find alternative strategies, such as tissue tolerance, which use Na^+^ sequestration and K^+^ homeostasis to achieve tolerance without compromising productivity [12].

The limitations of traditional approaches, which have largely focused on Na^+^ exclusion through transporters such as HKT1, combined with the need to increase rice production by 28% by 2050 to meet the growing demand [15], emphasize the urgent need for a paradigm shift in rice breeding for salinity tolerance and the discovery of new breeding targets. With climate models predicting an accelerated rate of land salinization, especially in coastal and low-lying areas, where groundwater salinization is a dominant process [16], there is an urgent need for a more comprehensive understanding of salt tolerance mechanisms. Rather than focusing on Na^+^ exclusion or ion transporters, future breeding strategies must address the complex interplay among osmotic regulation, ion homeostasis, and stress signaling pathways. In this study, we employed in silico modeling to investigate how key physiological traits—stomatal conductance (Gs), chlorophyll content (SPAD), shoot K^+^ content, and shoot Na^+^ content—might affect shoot dry weight of salt-grown rice plants. Our findings indicate that shoot Na^+^ content is not the best proxy for salinity stress tolerance, and its use in breeding programs may be counterproductive and misleading. Instead, our work suggests that enhancing K^+^ retention and optimizing stomatal development and operation may be a more effective strategy for improving rice growth under salinity. Overall, this work proposes new directions for breeding programs to ensure rice productivity and food security under changing climatic conditions.

## 2. Results

### 2.1. Selection of Regression Models

The modeling was done by using glasshouse data for six cultivated (*Oryza sativa* L.) and four wild rice genotypes (see Table 1 and Section 4 for details). The choice of parameters measured was driven by both their physiological relevance (e.g., importance of K/Na ratio for salinity tolerance) and their suitability for high-throughput screening (e.g., Gs and SPAD readings). In this context, other parameters such as root traits (e.g., root fresh or dry weight) or xylem sap ion content were excluded as far too labor- and time-consuming and thus not suitable as proxies in breeding programs. As all experiments were conducted at the vegetative stage of development, relative shoot dry weight (DW of salt-grown plants expressed as % of DW for control plants) was used as a proxy for tolerance. The raw values for all parameters measured that were used for modeling are shown in Table 1.

Stepwise Regression Analysis (SRA) was used to describe plant growth as a function of measured parameters (see Section 4). All regression models (at the consecutive steps of the analysis) predict the value of the dependent variable *Y*, the shoot dry weight (SDW) in g/plant, as a function of four independent variables:

- *X*_1_, the chlorophyll content (SPAD) in arbitrary units;
- *X*_2_, the stomatal conductance (Gs) in mmol·m^−2^/s;
- *X*_3_, the shoot sap Na concentration (shoot Na^+^) in mM;
- *X*_4_, the shoot sap K concentration (shoot K^+^) in mM.

The initial SRA model contained fifteen coefficients:

(1) $$Y=\beta_{0}+\sum_{i=1}^{4}\beta_{i}X_{i}+\sum_{i=1}^{4}\beta_{i,i}{\left(X_{i}\right)}^{2}+\sum_{i=1}^{3}\sum_{j=i+1}^{4}\beta_{i,j}X_{i}X_{j}+\varepsilon$$

The multiple linear regression (MLR) model (1) was constructed using 146 records containing the values of the independent and dependent variables. The modified stepwise regression algorithm (MSRA) finished in 11 steps (10 in phase one, 1 in phase two, and 0 in phase three):

(2) $$Y=\beta_{2}X_{2}+\beta_{4}X_{4}+\beta_{1,1}{\left(X_{1}\right)}^{2}+\beta_{2,2}{\left(X_{2}\right)}^{2}+\beta_{4,4}{\left(X_{4}\right)}^{2}+\varepsilon$$

The cycled outlier detection phase algorithm (CODPA) with six cycles was applied to model (2) once and identified 22 outliers (four in cycle one, six in cycle two, five in cycle three, two in cycle four, four in cycle five, and one in cycle six). The maximum allowed false-discovery rate for the Benjamini–Hochberg procedure in CODPA was set to 30%. The final model, constructed using the structure of (2) with the remaining 124 records, deemed by the SRA as best reflecting plant performance, was:

(3) $$\begin{array}{cc} Y= & 4.874\times 10^{-2}X_{2}+1.261\times 10^{-2}X_{4}-3.803\times 10^{-4}{\left(X_{1}\right)}^{2}-5.280\times 10^{-4}{\left(X_{2}\right)}^{2}-3.009\times 10^{-5}{\left(X_{4}\right)}^{2}+\varepsilon \\ & \left(4.91\times 10^{-3}\right)\;\;\left(1.46\times 10^{-3}\right)\;\;\;\;\left(9.46\times 10^{-5}\right)\;\;\;\;\;\;\;\;\left(5.39\times 10^{-5}\right)\;\;\;\;\;\;\;\;\left(5.81\times 10^{-6}\right)\;\;\left(0.280\right) \end{array}$$

The heteroskedasticity-consistent (HC3) estimates of the coefficients’ standard deviations are given in brackets under the coefficients in (3). The standard deviation of the residuals is shown under the term *ε* in (3). The robust HC3 covariance matrix, ***S***, of the five slopes in (3) is given on and above the diagonal in Table 2 (since it is symmetric).

The performance measures of the final regression model (3) are as follows:

- The root mean square error estimated with holdout residuals (*RMSE_HO_*) is 0.288;
- The mean absolute error estimated with holdout residuals (*MAE_HO_*) is 0.236;
- The adjusted coefficient of determination ($R_{adj}^{2}$) is 0.671;
- The coefficient of determination estimated with holdout residuals ($R_{HO}^{2}$) is 0.649.

The ANOVA test showed that model (3) is adequate with a *p*-value of 0.

The Expected Error Nullity Test yielded a *p*-value of 0.989, indicating insufficient evidence to reject the hypothesis that the expected error in the regression model is zero.

The modified heteroskedasticity testing and relaxing algorithm (MHTRA) produced a *p*-value of the ANOVA test for the full quadratic predicted residual model of 0.1913, indicating no statistically significant heteroskedasticity. Its adjusted coefficient of determination was 0.003, which, according to MHTRA, supports the homoskedasticity claim.

The modified Jarque–Bera Monte Carlo test for error normality was performed using 10,000 pseudo-realities. Since the *p*-value was 0.154, there was not enough statistical evidence to reject the normality of the error variable *ε* in (3).

A detailed description of the model in each step of the SRA, along with the data, is available in file *SM1.txt* in the Supplementary Materials.

The best regression model for SDW prediction included Gs, SPAD, and shoot K^+^ content, but not Na^+^ content, indicating that variation in the shoot Na^+^ content did not contribute significantly to biomass prediction. Gs and K^+^ content exhibited positive linear and negative quadratic effects, whereas SPAD showed a negative quadratic effect, with higher values associated with reduced SDW. Overall, the best regression model revealed that shoot biomass accumulation mainly depended on Gs, SPAD, and K^+^ content, whereas overall Na^+^ content played a less important role, thereby questioning the importance of Na exclusion as a key strategy for improving growth under salt stress.

### 2.2. Model Predictions for Fixed K^+^ and Variable SPAD and Gs

**Figure 1:**
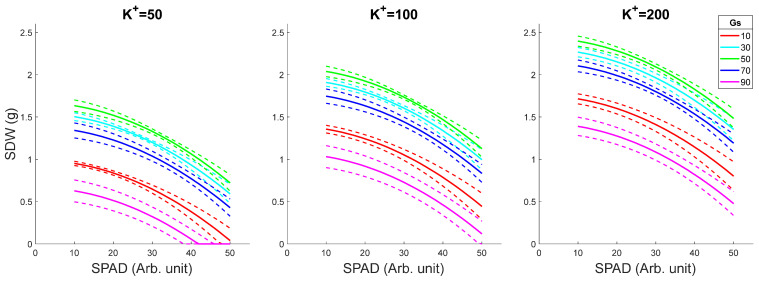
SDW as a function of SPAD for fixed K^+^ and variable Gs levels. The dashed lines in the figure depict 50% confidence intervals. K^+^ values are given in mM and Gs values in mmol·m^−2^/s.

We then asked the question of how changes in SPAD value might impact plant growth (SDW) for a range of fixed K^+^ concentrations and as a function of changing Gs values. As shown in Figure 1, the in silico prediction showed that the SDW decreases slightly along with an increase in SPAD value (from 10 to 50, an expected physiological range), indicating that photochemical limitation was not the primary reason for growth inhibition under saline conditions in rice; this trend may also reflect a “condensation effect”. The latter implies that reduced cell size in salt-grown plants may increase the density of chlorophyll per surface unit. Regardless of K^+^ availability, the optimal plant performance was observed for Gs values of 50 mmol·m^−2^/s (green lines in each panel). This trend remains the same when the shoot K^+^ content is set at three levels (50/100/200 mM) with SDW being directly proportional to K^+^ content in the leaf sap. Interestingly, an increase in Gs values over 50 mmol·m^−2^/s was counterproductive to plant performance (Figure 1), most likely due to excessive water loss.

### 2.3. Model Predictions for Fixed SPAD and Variable Gs and K^+^ Levels

**Figure 2:**
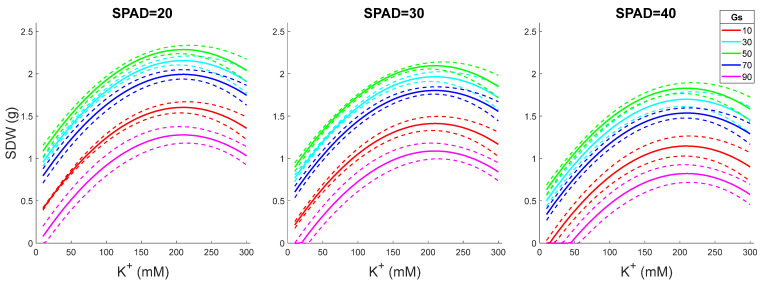
SDW as a function of leaf K^+^ for fixed SPAD and variable Gs values. The dashed lines in the figure depict 50% confidence intervals. SPAD values are given in arbitrary units and Gs values in mmol·m^−2^/s.

**Figure 3:**
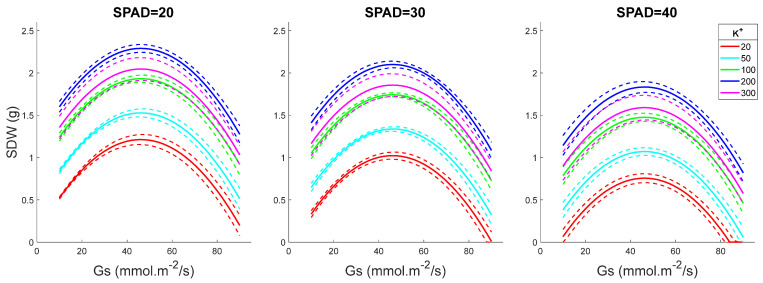
SDW as a function of Gs for fixed SPAD and variable K^+^ values. The dashed lines in the figure depict 50% confidence intervals. SPAD values are given in arbitrary units and K^+^ values in mM.

Shoot DW increased with increasing leaf K^+^ content, peaking at around 220 mM K^+^ in the leaf sap (Figure 2). This trend was observed for all SPAD values, with the optimal performance reported for Gs = 50 mmol·m^−2^/s, regardless of SPAD readings. The highest performance was predicted for Gs = 50, SPAD = 20, and K^+^ = 200 mM. The same predictions were obtained by plotting SDW vs. Gs values (Figure 3).

### 2.4. Model Predictions for Fixed K^+^ and Variable SPAD and Gs Levels

**Figure 4:**
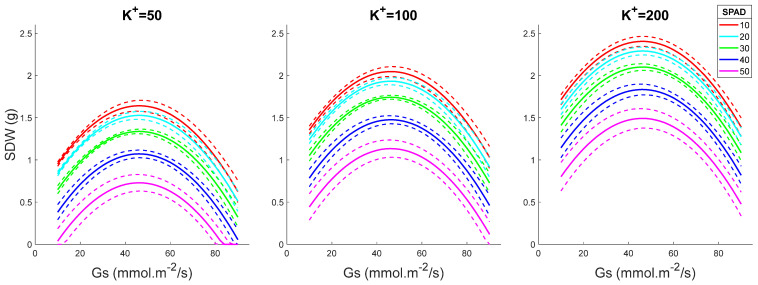
SDW as a function of Gs for fixed K^+^ and variable SPAD levels. The dashed lines in the figure depict 50% confidence intervals. SPAD values are given in arbitrary units and K^+^ values in mM.

**Figure 5:**
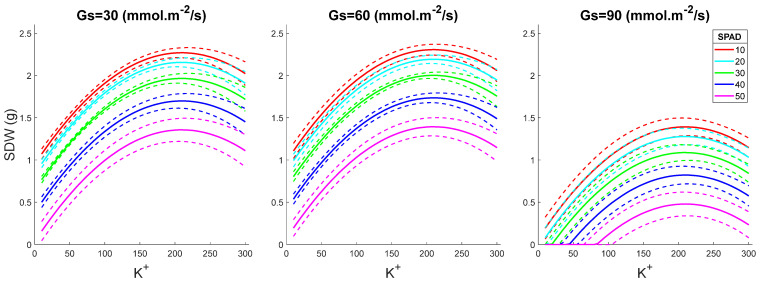
SDW as a function of K^+^ for fixed Gs and variable SPAD levels. The dashed lines in the figure depict 50% confidence intervals. SPAD values are given in arbitrary units and Gs values in mmol·m^−2^/s.

Shoot DW increased with increasing Gs values, peaking at 50 mmol·m^−2^/s and declining at higher stomatal conductance (Figure 4). The higher the shoot K^+^ was, the stronger the plant performance (SDW value). Surprisingly, SPAD values correlated negatively with plant performance, indicating again that chlorophyll content cannot be used as a reliable proxy for salinity tolerance screening. The same conclusions were made by plotting SDW as a function of K^+^ for fixed Gs levels and for various SPAD readings (Figure 5).

### 2.5. Overall Summary and the Best Set of Parameters

Overall, the in silico model predictions revealed that SDW is mainly determined by Gs, SPAD, and shoot K^+^ content. SDW decreased consistently with increasing SPAD when Gs and K^+^ content were fixed, and this trend was observed across different K^+^ and Gs levels. When Gs varied, SDW showed a parabolic response with a peak at around 45 mmol·m^−2^/s, while SDW also exhibited a similar parabolic relationship with an optimum at approximately 220 mM under variable K^+^ conditions. These results suggest that the best conditions for maximizing SDW involve a moderate stomatal conductance (around 45 mmol·m^−2^/s), an optimal shoot K^+^ content (around 220 mM), and relatively low SPAD values. This finding highlights the importance of achieving a balanced coordination among gas exchange, nutrient accumulation, and chlorophyll content to enhance biomass production under salt stress.

## 3. Discussion

### 3.1. Targeting Na^+^ Exclusion from Uptake May Be Counterproductive

For many decades, the focus on improving salt tolerance in plants (including rice) was on minimizing Na^+^ content by enhancing its exclusion, a process mediated by *HKT* transporter genes. Several members of the HKT family in rice were characterized as Na^+^-selective transporters [9], and OsHKTA1;5 was identified as a key transporter responsible for unloading Na^+^ from xylem sap in roots [10], a crucial mechanism for protecting shoots from Na^+^ toxicity. It was also shown that natural variation in *OsHKTA1;5*, including specific amino acid substitutions, may contribute to differential salt tolerance between indica and japonica subspecies [17]. Beyond OsHKTA1;5, OsHKT1;1 also plays a role in limiting Na^+^ accumulation in shoots, as evidenced by the increased salt sensitivity and Na^+^ over-accumulation observed in *OsHKT1;1* knockout mutant [18]. Surprisingly, genome-wide association studies of biomass and tissue ion content in 176 rice landraces from Bangladesh have revealed 13 novel QTLs, but none of them were related to HKT transporters [19]. Using genomic resequencing analysis of a salt-tolerant introgression line derived from a population of the salt-tolerant wild rice line Dongxiang (*O. rufipogon* Griff.) hybridized with a cultivated rice variety (*O. sativa* ssp. *japonica*), Ningjing16, Quan et al. [20] located in the qST1.2 region the gene that was previously defined as *OsHKT1;5* [10]. However, years later, no evidence is available in the literature suggesting that targeting this gene may result in a significant improvement of salinity tolerance in rice. Consistent with this, in this study, the regression model suggests that Na^+^ content is less important (compared to SPAD, Gs, and shoot K^+^) in contributing to shoot dry matter, indicating that targeting Na^+^ exclusion in rice for boosting salt tolerance may not be the best option. While we acknowledge that these conclusions are based on a limited number of genotypes and subspecies and need to be validated in future experiments (e.g., including both *japonica* and *indica* subspecies, taking plants to grain yield, etc.), it nonetheless calls for a paradigm shift in rice breeding.

Na^+^ exclusion comes with two caveats, both related to high energy cost. Na^+^ exclusion from uptake by roots is mediated by the *SOS1* Na^+^/H^+^ exchanger, which requires H^+^-ATPase to consume ATP to restore the proton gradient across the plasma membrane. It is estimated that in cereals up to 95% of all Na^+^ taken by the root is extruded back to the rhizosphere via this mechanism [21,22]. In combination with the existence of the bypass flow in rice roots [23] that allows Na^+^ uptake via the apoplastic pathway to be loaded into the xylem, such a mechanism results in a futile cycle and energy waste. Equally counterproductive is the reliance on Na^+^ retrieval from the shoot mediated by HKT1 transporters. To be able to grow under hypersaline conditions, plants need to maintain a positive turgor pressure. This requires the osmotic adjustment and, in the absence of Na^+^ as a “cheap osmoticum”, plants need to synthesize organic osmolytes to counter the salt-induced osmotic stress [24,25]. The biosynthesis of compatible solutes such as proline and glycine betaine typically consumes hundreds of kilojoules per mole (ca. 400–1000 kJ mol^−1^), mainly through ATP- and NADPH-dependent reactions [13]. By comparison, using inorganic ions (such as Na^+^) for osmotic adjustment costs an order of magnitude less energy (<100 kJ mol^−1^), highlighting why ion retention and compartmentation are more important than organic solute accumulation under salt stress. Thus, instead of excluding Na^+^, plants may be much better at using it as a more energy-efficient osmolyte. This is only possible on the condition that accumulated Na^+^ does not interfere with metabolic processes in the cytosol, implying efficient vacuolar Na^+^ sequestration. A representative example is the halophytic rice *Oryza coarctata*, which rapidly increases xylem Na^+^ loading to promote osmotic adjustment, thereby maintaining leaf water status under salt stress [26]. Moreover, *O. coarctata* maintains cellular function despite this substantial Na^+^ flux by upregulating vascular NHX-type antiporters and V-ATPase/V-PPase proton pumps [27], thereby generating the proton motive force required for efficient Na^+^ sequestration into vacuoles [28] and preventing cytosolic toxicity. This coordinated enhancement of Na^+^ inclusion and vacuolar compartmentation enables the species to tolerate elevated apoplastic Na^+^ concentrations without compromising cellular function. Consequently, *O. coarctata* exhibits superior salt tolerance through a strategy focused on Na^+^ inclusion and compartmentalization, rather than relying on the more energy-costly process of Na^+^ exclusion. However, the traditional analysis of shoot Na^+^ content by flame photometry or ICP methods does not discriminate between vacuolar and cytosolic distribution of Na^+^, making this parameter misleading and unsuitable as a proxy for screening programs. The fact that shoot Na^+^ content was the least important of four predictive parameters in our models fully supports this notion.

### 3.2. Why May K^+^ Retention Be More Important than Na^+^ Exclusion?

Although Na^+^ exclusion has traditionally been regarded as a key factor of salt tolerance, our findings suggest that K^+^ retention plays an even more critical role in promoting growth and biomass accumulation under saline conditions in rice. K^+^ is essential not only for maintaining osmotic balance and cellular integrity but also for supporting efficient photosynthesis, enzymatic activity, and signal transduction during salt stress. K^+^ is crucial for plant adaptation under salinity, as it is involved in a wide range of physiological processes essential for both growth and stress tolerance [29]. This is further supported by evidence showing that exogenous potassium supplementation improves wheat biomass, photosynthetic pigment content, osmolyte accumulation, and antioxidant enzyme activities, thereby alleviating oxidative damage induced by salt stress [30,31,32].

In this study, when SPAD and Gs were fixed, the predicted SDW increased with K^+^ content up to 220 mM (Figure 5), highlighting the importance of shoot accumulation and retention in the salt-grown rice plants. The physiological rationale may be as follows. First, K^+^ is a key component of the chloroplast machinery, regulating photosynthetic electron transport and the activity of the ATP synthase complex [33]. For example, K^+^ is indispensable for modulating the thylakoid proton motive force (pmf) and enables charge compensation across the membrane. This occurs through K^+^ channels, such as TPK3. This mechanism minimizes the transient proton diffusion potential generated when protons exit the lumen via ATP synthase, thereby accelerating proton efflux and enhancing ATP synthesis [33]. Under salt stress, the loss of stromal K^+^ disrupts this balance, leading to reduced ATP production, impaired carbon assimilation and over-reduction of the photosynthetic electron transport chain. It also promotes ROS formation, resulting in oxidative damage to the photosynthetic apparatus and triggering retrograde signaling that downregulates photosynthesis-related genes. This notion is supported by observations of damaged chloroplast ultrastructure and decreased photosynthetic performance in K-sensitive maize genotypes [34,35]. Potassium also plays a key role in maintaining thylakoid membrane structure and stability by regulating the ionic environment within the stroma and lumen, thereby preventing membrane swelling, grana unstacking and impairment of PSII; mechanistic and physiological studies show that K^+^/H^+^ transport and K^+^ channels modulate pmf components and protect thylakoid function under salt stress [34,36,37,38]. It is also critical for efficient light absorption and energy conversion during photosynthesis. Silencing the thylakoid-localized *TPK3 K^+^* channel gene in *Arabidopsis* disrupts photosynthesis and thylakoid function [39]. Under salt stress, excessive Na^+^ accumulation damages chloroplast integrity and reduces photosynthetic efficiency, whereas adequate K^+^ levels help preserve the functionality of the photosynthetic apparatus, which is vital for carbon fixation and biomass production. In addition, K^+^ plays a crucial role in osmotic adjustment by maintaining cell turgor and preventing dehydration under saline conditions [21]. Recent studies have emphasized that this protective effect relies on maintaining a high cytosolic K^+^/Na^+^ ratio, which is a key determinant of salt tolerance [40]. Excessive Na^+^ accumulation can induce oxidative stress and trigger programmed cell death (PCD), a process in which K^+^ loss is a critical initiating factor [29]. Beyond its role in ionic balance, K^+^ also acts as an essential cofactor for many enzymes involved in core metabolic pathways, including carbohydrate and protein synthesis. For example, K^+^ enhances the activity of ATPase associated with the chloroplast inner envelope, an enzyme crucial for regulating stromal ion balance and sustaining photosynthetic carbon fixation [41].

Beyond its structural and metabolic roles, K^+^ is also important for plant signaling and has been suggested as a new second messenger mediating plant adaptive responses to salinity [29]. Under salt stress, K^+^ acts as a crucial signaling molecule that interacts with the core Ca^2+^ and ROS signaling pathways to regulate plant adaptation [42]. K^+^ is also vital for stomatal function, as the stomatal opening and closing rely on K^+^ uptake and loss for adjusting the turgor pressure of the guard cells, respectively [43,44]. Beyond its osmotic function, K^+^ is important for systemic signaling, as evidenced by the coordination of stomatal behavior across epidermal cell types via the AtKC1 channel subunit [45].

### 3.3. What Genes Should Be Targeted to Optimize Gs and K^+^ Content?

Salinity stress significantly impacts stomatal development in cultivated rice, resulting in a smaller stomatal size and a decreased proportion of stomata on the leaf epidermis, consequently leading to a decrease in Gs by 68%, causing stomatal limitation in the photosynthetic process [46]. When SPAD and K levels are fixed, the predicted SDW reaches a peak at the optimal Gs level and decreases when Gs is lower or higher than that level (Figure 2). Although the exact value for the optimal Gs may differ under different conditions, this trend seems plausible as low Gs will limit the CO_2_ supply, which restrains photosynthesis, and high Gs will increase transpiration, which will not only increase Na^+^ loading but also decrease the leaf water potential. Several genes and transcription factors (TFs) are involved in stomatal development in rice, such as *EPIDERMAL PATTERNING FACTOR1 (OsEPF1)*, *SPEECHLESS (OsSPCH)*, *INDUCER OF CBF EXPRESSION (OsICE)*, and *OsMUTE*, mediating initiation of stomatal lineage and guard mother cell transition [47,48]. By overexpressing *OsEPF1*, the rice plants exhibited reduced stomatal density and enhanced tolerance to drought and heat stress, due to improved water conservation [49]. The same overexpression line also showed less Na^+^ accumulation in the leaf under 20 mM NaCl conditions [50]. The ABA signal plays a crucial role in regulating stomatal closure under salt conditions [51,52,53]. Some halophytes, such as wild rice *O. coarctata*, are insensitive to exogenous ABA compared to cultivars, which confers stable Gs when growing under salt conditions [46]; halophyte *Thellungiella salsuginea* showed unaffected Gs due to low ABA content in leaves and suppressed ABA receptor genes (*PYL1,4,5,6*) under salt conditions [54]. Interestingly, a recent study in *Arabidopsis* showed that sodium-specific gene expression and phenotypic response in the root are due to a delayed ABA response, despite the fact that endogenous ABA is required for sodium stress adaptation [55]. Hence, it is plausible to target the above TFs or genes to have reduced stomatal density and ABA-insensitive stomatal operation (more stable Gs) phenotypes in cultivars under salt conditions.

K^+^ retention is crucial for salt stress adaptation [29]. In cultivated rice, xylem K^+^ concentration was reduced 2.5-fold after 5 days of 50 mM NaCl treatment [56]. This is consistent with the model prediction in the present work, which predicted SDW decreases along with a decline in K^+^ while SPAD and Gs are fixed (Figure 2). Under salt conditions, excessive Na^+^ enters the cytosol from the apoplast via Non-Selective Cation Channels (NSCCs) passively [57], resulting in depolarization of the plasma membrane (PM) and subsequently activating the voltage-gated Guard Cell Outward Rectifying K^+^ (GORK) channel for K^+^ efflux [29]. In cultivated rice, the NaCl-induced K^+^ loss from mesophyll cells also occurs via NSCCs [58]. In addition to increasing Na^+^ extrusion (which is likely futile under high salt conditions), maintaining a more negative plasma membrane potential (MP) and reducing the sensitivity of K^+^-permeable channels to ROS are two predominant strategies for improving K^+^ retention and salt tolerance. Plasma membrane potential (MP) is predominantly maintained by H^+^-ATPase (encoded by AHA1), which pumps out cytosolic H^+^ [59,60]. Alternatively, the tonoplast-localized ATPase and PPase (encoded by VHA1 and AVP1, respectively) pump cytosolic protons into the vacuole, thereby helping maintain negative MP. But all these processes require energy. A recent study demonstrated that halophytic rice (*O. coarctata*) can maintain a hyperpolarized plasma membrane in mesophyll cells under salt conditions in a chloride-dependent manner [54], although the involved transporter remains to be revealed. The PM hyperpolarization could suppress K^+^ loss from GORK and induce K^+^ uptake via AKT1 [61]. In addition, the HAK5 could also contribute to this process [62].

The critical role of cytosolic K^+^ as a key determinant of salinity tolerance in plants has been demonstrated for many plant species (see [29] for review). For example, Chen et al. [63] screened 70 barley genotypes and demonstrated a very strong (R2 = 0.62) correlation between their overall salinity tolerance (judged by relative biomass and/or grain yield) and the ability of plant roots to retain K+ upon salinity exposure. Another example is [64]. These authors performed a GWAS analysis for root Na^+^/K^+^ ratio in a population consisting of 369 tomato accessions and found that a variation in the coding sequence of SlHAK20 (high-affinity K^+^ transporter from the HAK/KUP family) was found to confer salt tolerance in tomato plants. Also, knockout mutations in tomato *SlHAK20* and the rice homologous genes resulted in hypersensitivity to salt stress. These and other studies provide strong evidence that targeting mechanisms enabling more efficient K^+^ uptake and/or retention may be a highly promising approach to improve salinity stress tolerance in crops. Importantly, as demonstrated by [64], this ability was present in wild crop relatives but was lost during domestication.

Abiotic stress tolerance was present in wild relatives but lost during domestication as a result of targeting yield- and quality-related traits [65,66,67]; this is specifically true for salt stress. While it is widely recognized that such traits should be regained [68], the focus of plant breeders was mostly on salt exclusion mechanisms. As shown in this work and argued elsewhere [12], such an approach is counterproductive and can explain the lack of any significant breakthrough in creating salt-tolerant rice varieties. This calls for a major paradigm shift and a need for targets that appear to be more critical to plant adaptation to hypersaline environments.

## 4. Materials and Methods

### 4.1. Plant Species and Growth Conditions

The current work is the extension of the previously published paper [69] utilizing a subset of raw data for modeling. Overall, six cultivated varieties of rice (*Oryza sativa* L.) and four wild rice genotypes were used for modeling (Table 1). Seeds were obtained from the International Rice Research Institute (IRRI). The experiment was conducted in 2018–2019 (from October to January) under the glasshouse conditions at the Sandy Bay campus of the University of Tasmania (Hobart).

The full details on the experimental design, treatments, as well as statistical analysis can be found in [69]. In brief, plants were cultivated in pots using standard potting mixture comprising 80% composted pine bark, 10% each of sand and coir peat, plus slow-release fertilizer (N:P:K ratio of 8:4:10). Seeds of different rice genotypes were directly sown in plastic trays, which were kept in the room at a temperature of 30 °C for germination and seedling establishment. Seven-day-old rice seedlings were transplanted into 1.5 L pots (5–6 plants per pot) and grown in a glasshouse under controlled temperature (26–28 °C) for two weeks to establish seedlings, before salinity stress was applied. For the latter, three salt levels (added with irrigation water on a daily basis) were used: (1) control (no salt); (2) moderate stress, 50 mM NaCl; and (3) severe salt stress, 100 mM NaCl. Plants were treated with salt for three weeks.

### 4.2. Physiological Measurements

#### 4.2.1. Chlorophyll Contents and Stomatal Conductance

Measurements for chlorophyll content (SPAD) and stomatal conductance (Gs) were performed on the youngest fully expanded leaf at the end of the experiment, i.e., 21 days after treatment (DAT). The relative chlorophyll content in leaves was measured by SPAD readings using a SPAD-502 meter (Konica Minolta, Osaka, Japan). All measurements were taken from the region located about one-third of the way from the leaf tip. Eight to ten random recordings were taken and averaged to constitute one replicate; 10 replicates were measured for each treatment. For Gs measurements, the Decagon Leaf Porometer (Decagon Devices Inc., Pullman, WA, Australia) was used to measure the Gs value under full daylight conditions between 10:00 and 12:00 h. Up to ten individual replicates were taken from each treatment.

#### 4.2.2. Fresh and Dry Plant Biomass

Plants were harvested after three weeks of salt treatment, and the shoot fresh weight (SFW) per plant was determined immediately by using an analytical balance. Fresh samples were then placed in paper bags and oven-dried at 70 °C for 72 h to achieve a constant dry weight for measuring shoot and root dry weights.

#### 4.2.3. Tissue Na^+^ and K^+^ Content

Na^+^ and K^+^ content in the shoot was quantified using leaf sap extracts following the standard procedure established in our laboratory [54]. Shoot and root ion contents (Na^+^ and K^+^) were then determined using a flame photometer (PFP7, Jenway; Bibby Scientific Ltd., Stone, UK).

### 4.3. Modeling Procedures

The values of the four independent variables described in Section 2.1 were organized into a 4-dimensional vector of independent input $\vec{X}={\left(X_{1},X_{2},X_{3},X_{4}\right)}^{T}$. Then, the MLR model (1) can be equivalently represented as $Y=E\left(Y|\vec{X}\right)+\varepsilon$, where *Y* is the random variable SDW, ε is the random error term, and $E\left(Y|\vec{X}\right)$ is the expected value of *Y* at a given vector of independent input $\vec{X}$. Overall, the data consisted of several replicates for each of the 30 combinations of species (see Table 1) and salinity stress (see Section 4.1) and was organized in a data set containing *n* = 146 records, $\left\{\langle {\vec{x}}_{i},y_{i}^{mes}\rangle ,\;i=1,2,\ldots ,n\right\}$, where ${\vec{x}}_{i}$ and $y_{i}^{mes}$ stand for the specific values of $\vec{X}$ and Y in record i. The constructed model (1) using the data set contained 15 coefficients/slopes, yet only some were statistically significant. Model (2) was constructed by applying the modified stepwise regression algorithm (MSRA) from [70] to identify the set of regressors with maximum cardinality, containing only significant slopes. The slopes of the MLR_k_ (i.e., the MLR model at step *k* of MSRA) were estimated using singular value decomposition [71] with corrected singular values as shown in [72]. In compliance with [73], the predicted residuals from MLR_k_ were estimated and used to compute the following holdout performance measures of the regression: RMSE_HO,_ MAE_HO_, and $R_{HO}^{2}$. The predicted residuals were also used to compute the standard deviations of the computed slopes by applying the robust, unbiased, heteroscedasticity-consistent HC3 estimator [74] of the model parameters’ covariance matrix. Similarly, the nullity assumption (i.e., the mean value of the error variable is zero) was confirmed by Student’s *t*-test based on the predicted residuals. The modified heteroskedasticity testing and relaxing algorithm (MHTRA), described in [70], was utilized to test heteroskedasticity in MLR_k_. MHTRA combines the ideas in [75] with those in [76]. The normality assumption (i.e., that the error variable is normally distributed) was tested by a Monte Carlo version of the Jarque–Bera test [77] (pp. 131–132). The outliers in MLR (2) were identified using a 6-cycled version of Algorithm 2, called CODPA in [72]. The purged data containing *n* = 224 records was used to build the final model (3) using the above-described procedures for constructing MLR_k_.

### 4.4. Prediction with the Selected Model

With the selected model, we performed shoot dry weight (SDW) prediction in different scenarios (Figure 1, Figure 2, Figure 3, Figure 4 and Figure 5):

(1) Various SPAD values with fixed K^+^ and Gs levels (Figure 1): SPAD range from 10 to 50, interval gap of 1; K^+^ levels as 50, 100, and 200 mM; Gs levels as 10, 30, 50, 70, and 90 mmol·m^−2^/s.
(2) Various K^+^ values with fixed SPAD and Gs levels (Figure 2): K^+^ range from 10 to 300 mM, interval gap of 5; SPAD levels as 20, 30, and 40; Gs levels as 10, 30, 50, 70, and 90 mmol·m^−2^/s.
(3) Various Gs values with fixed SPAD and K^+^ levels (Figure 3): Gs range from 10 to 90 mmol·m^−2^/s, interval gap of 2; SPAD levels as 20, 30, and 40; K^+^ levels as 20, 50, 100, 200, and 300 mM.
(4) Various Gs values with fixed K^+^ and SPAD levels (Figure 4): Gs range from 10 to 90 mmol·m^−2^/s, interval gap of 2; K^+^ levels as 50, 100, and 200 mM; SPAD levels as 10, 20, 30, 40, and 50.
(5) Various K^+^ values with fixed Gs and SPAD levels (Figure 5): K^+^ range from 10 to 300 mM, interval gap of 5; Gs levels as 30, 60, and 90 mmol·m^−2^/s; SPAD levels as 10, 20, 30, 40, and 50.

To construct Figure 1, Figure 2, Figure 3, Figure 4 and Figure 5, we predict $E\left(Y|\vec{x}\right)$ and $V\left(Y|\vec{x}\right)$, i.e., the mean and the variance of the predicted value *Y* = SDW at given variables organized in the five-dimensional column observation vector $\vec{x}={\left({\mathrm{Gs},\;K}^{+},\;{\left(\mathrm{SPAD}\right)}^{2},\;{\left(\mathrm{Gs}\right)}^{2},\;{\left(K^{+}\right)}^{2}\right)}^{T}$. The mean, $E\left(Y|\vec{x}\right)$, was calculated by (3) without the last term, ε. The variance, $V\left(Y|\vec{x}\right)$, was calculated using the formula $V\left(Y|\vec{x}\right)={\vec{x}}^{T}S\vec{x}$ (see [77] (pp. 861–864)). In the last formula, the covariance matrix of the slopes ***S*** was substituted with its robust HC3 estimate, given in Table 2. The 50% confidence interval of *Y* given $\vec{x}$ was estimated assuming that the predicted SDW value is *t*-distributed with ν=124−5=119 degrees of freedom.

The data for the figures is available in the file *SM2.txt* in the Supplementary Materials.

The count of outliers in model (3) was 22, which amounts to less than 15% of the 146 available data points. Some researchers claim that outliers should not be removed from the data. The model without outlier removal constructed with all 146 data points is available in the file *SM1.txt* in the Supplementary Materials. Figures similar to those in Figure 1, Figure 2, Figure 3, Figure 4 and Figure 5 calculated using this model are available in the file *SM4.pdf* in the Supplementary Materials with the data from the file *SM3.txt*. Also, the qualitative conclusions reached in the paper do not depend on outlier removal.

### 4.5. Software

All calculations in the paper are supported through original software, developed in MATLAB R2024b©. Readers can obtain the software free of charge upon request from the authors.

## 5. Conclusions

This study challenged the classical notion that salinity tolerance in rice is determined by its ability to reduce shoot Na^+^ content. Instead, using a predictive in silico model, we demonstrate that plant growth under saline conditions is primarily determined by stomatal conductance (Gs) and shoot K^+^ content. The poor predictive value of shoot Na^+^ is explained by its inability to adequately account for intracellular Na^+^ sequestration by analyzing tissue Na^+^ content and is explained in the context of the energy cost of plants’ osmotic adjustment. It is concluded that targeting Na^+^ exclusion traits in rice breeding appears to be counterproductive, and future efforts should focus on tissue tolerance mechanisms, specifically K^+^ retention in mesophyll—complemented by optimal stomatal operation. Targeting these traits may develop rice genotypes suitable for future climate scenarios.

## Figures and Tables

**Table 1:** Plant materials used in the study.

Type	Species Name/Cultivar
Wild	*Oryza alta*
Wild	*Oryza barthii*
Wild	*Oryza australiensis*
Wild	*Oryza punctata*
Cultivated	*Oryza sativa* cv H-86
Cultivated	*Oryza sativa* cv Pusa Basmati
Cultivated	*Oryza sativa* cv Pokkali
Cultivated	*Oryza sativa* cv Nipponbare
Cultivated	*Oryza sativa* cv IR29
Cultivated	*Oryza sativa* cv IR1

**Table 2:** Robust HC3 covariance matrix of the slopes in (3). The correlation coefficients are shown under the main diagonal. The indexing of the slopes follows Equation (2).

	$\beta_{2}$	$\beta_{4}$	$\beta_{1,1}$	$\beta_{2,2}$	$\beta_{4,4}$
$\beta_{2}$	2.411 × 10^−5^	−5.088 × 10^−6^	−3.172 × 10^−7^	−2.381 × 10^−7^	1.764 × 10^−8^
$\beta_{4}$	−0.7091	2.135 × 10^−6^	1.585 × 10^−8^	5.244 × 10^−8^	−8.001 × 10^−9^
$\beta_{1,1}$	−0.6832	0.1147	8.942 × 10^−9^	2.227 × 10^−9^	−1.135 × 10^−11^
$\beta_{2,2}$	−0.8992	0.6655	0.4368	2.908 × 10^−9^	−2.118 × 10^−10^
$\beta_{4,4}$	0.6180	−0.9419	−0.02065	−0.6757	3.380 × 10^−11^

## Data Availability

The original contributions presented in this study are included in the article/Supplementary Materials. Further inquiries can be directed to the corresponding author.

## Supplementary Materials

The following supporting information can be downloaded at: https://www.mdpi.com/article/10.3390/plants15040597/s1.

## Abbreviations

The following abbreviations are used in this manuscript:

Gs	Stomatal conductance
SDW	Shoot dry weight
PCD	Programmed cell death
pmf	Proton motive force
PM	Plasma membrane
