# Supplementary material for: Are We Chasing a Wild Goose? Rethinking Breeding Targets for Salinity Stress Tolerance in Rice
Source: Plants (Basel). 2026 Feb 13;15(4):597. doi: 10.3390/plants15040597 (PMC12943982; doi:10.3390/plants15040597)
Supplement: Supplementary file 1 [file plants-15-00597-s001.zip › SM4.docx]

Figure 1

Figure 2

Figure 3

Figure 4

Figure 5
